# Impact of Maternal Overweight and Obesity on Adipokines During Pregnancy and Lactation

**DOI:** 10.3390/ijms26199757

**Published:** 2025-10-07

**Authors:** Anita Froń, Paulina Tomecka, Magdalena Orczyk-Pawiłowicz

**Affiliations:** 1 Division of Chemistry and Immunochemistry, Department of Biochemistry and Immunochemistry, Wroclaw Medical University, M. Skłodowskiej-Curie 48/50, 50-369 Wroclaw, Poland; 2 University Clinical Hospital, Borowska 213, 50-556 Wroclaw, Poland; tomeckapaulina@gmail.com

**Keywords:** overweight, obesity, BMI, adipokines, pregnancy, lactation, breast milk

## Abstract

Maternal overweight and obesity have reached global epidemic levels, altering metabolic adaptations during pregnancy and lactation. Beyond their well-known impact on gestational outcomes, elevated BMI profoundly influences the secretion of adipokines—hormones derived from adipose tissue that circulate in maternal blood and are secreted into breast milk—thereby directly linking maternal metabolism to offspring development. In this state-of-the-art narrative review, we synthesize current evidence on how maternal overweight and obesity shape concentrations of key adipokines (leptin, adiponectin, ghrelin, obestatin, and resistin) in serum, cord blood and breast milk. Excess maternal weight robustly increases leptin, while effects on adiponectin, ghrelin, obestatin, and resistin remain uncertain. To our knowledge, this is the first review to focus specifically on the impact of maternal overweight and obesity on adipokine alterations across both pregnancy and lactation. Future studies should apply standardized sampling and analytical protocols and use longitudinal designs including body composition assessments to clarify their role in maternal and child metabolic health.

## 1. Introduction

Maternal overweight and obesity are increasingly prevalent worldwide and are associated with adverse outcomes for both mother and child [1]. Approximately one in five pregnant women (20%) currently live with obesity, and this figure is projected to rise to 23.3% by 2030 [2]. These trends are of particular concern given the well-established associations between excessive maternal body weight and increased risks of gestational diabetes mellitus (GDM), preeclampsia, cesarean delivery, and long-term cardiometabolic disease in both mother and child [3,4,5].

Adipose tissue is a metabolically active endocrine organ that secretes a wide range of adipokines. Current evidence indicates that the number of identified adipokines exceeds 600, excluding fatty acids and other non-peptidic metabolites [6,7].

In the context of overweight and obesity, adipose tissue undergoes significant metabolic and structural remodeling—including adipocyte hypertrophy, increased macrophage infiltration into adipose tissue, and formation of crown-like structures around dying cells. These changes establish a state of chronic low-grade inflammation, characterized by the secretion of pro-inflammatory cytokines such as TNF-α and IL-6, which impair insulin signaling and promote systemic insulin resistance [8,9,10,11].

Beyond their role in energy metabolism, adipokines are increasingly recognized as critical mediators linking maternal nutritional status to reproductive, vascular, and immune adaptations during pregnancy. Key adipokines such as leptin, adiponectin, resistin, visfatin, and chemerin are involved in the regulation of placental growth, trophoblast invasion, angiogenesis, and maternal–fetal nutrient transfer. They also modulate insulin sensitivity and glucose homeostasis, thereby shaping maternal metabolic adaptations that ensure an adequate nutrient supply for the fetus [8,12,13,14]. At the immunological level, adipokines influence the delicate balance between pro- and anti-inflammatory signaling, contributing to maternal immune tolerance of the fetus while simultaneously maintaining host defense [15,16,17]. During lactation, the presence of adipokines in breast milk provides a direct postnatal route of exposure for the infant. This transfer is increasingly regarded as a key mechanism of early-life metabolic programming, influencing appetite regulation, insulin sensitivity, and inflammatory responses in the offspring [5,18,19,20]. Alterations associated with maternal overweight and obesity may disrupt these finely tuned hormonal adaptations that are essential for supporting fetal development and lactation, thereby increasing the vulnerability of both mother and child to short- and long-term metabolic disorders [3,5,21,22]. These observations are consistent with the Developmental Origins of Health and Disease (DOHaD) paradigm, which posits that maternal metabolic and endocrine signals during pregnancy and early infancy have long-lasting consequences for the child’s risk of obesity, insulin resistance, and cardiometabolic disease later in life [23,24,25,26].

Despite growing interest in the endocrine function of adipose tissue, the specific alterations in adipokine profiles induced by maternal overweight and obesity during pregnancy and lactation remain incompletely understood. Clarifying these mechanisms may provide valuable insight into the developmental origins of metabolic disease in offspring. Although previous reviews have addressed adipokines in pregnancy or breast milk separately, none have specifically focused on the impact of maternal overweight and obesity across both stages [12,20,27,28]. Accordingly, this narrative review synthesizes evidence on how maternal overweight and obesity affect concentrations of key adipokines—leptin, adiponectin, ghrelin, obestatin, and resistin—across maternal serum, umbilical cord blood, and breast milk during pregnancy and lactation. It also discusses the implications for both clinical practice and future research. To our knowledge, this review is the first to place particular emphasis on maternal adiposity as a determinant of adipokine alterations during pregnancy and lactation.

An overview of reported alterations in circulating and milk-derived adipokines throughout pregnancy and lactation is presented in Table 1.

## 2. Pregnancy and Lactation in Overweight and Obesity

Pregnancy triggers extensive physiological adaptations across nearly all maternal organ systems. Early in gestation, the placenta, primarily of fetal origin, invades the uterine decidua and secretes a variety of hormones and signaling molecules that profoundly influence maternal physiology [55]. A notable adaptation is physiological hepatomegaly, which correlates positively with maternal weight gain and fetal number. This liver enlargement begins after implantation, peaks at delivery, and is associated with increased levels of IL-6, TNF-α, IL-1β, c-JUN, and activation of hepatic signaling pathways, including STAT3, β-catenin, and the epidermal growth factor receptor [56].

During pregnancy, total glucose production and gluconeogenesis increase to meet the growing energy requirements of the developing fetus. Concurrently, maternal insulin resistance develops, promoting hepatic glucose output, enhancing transport of fatty acids, triglycerides, and cholesterol, and stimulating protein synthesis from amino acids, while decreasing glucose and urea production from amino acid substrates. Leptin contributes to glucose homeostasis and hepatic metabolism by increasing phosphorylation of acetyl-CoA carboxylase, fatty acid oxidation, and ketogenesis [57]. Regardless of weight gain, liver size increases in most women during pregnancy and returns to baseline after weaning [58].

Significant cardiovascular remodeling occurs in pregnancy, characterized by elevated cardiac output, expanded extracellular fluid volume, reduced blood pressure and peripheral resistance [59]. A reversible form of eccentric hypertrophy, termed pregnancy-induced cardiac hypertrophy, develops in the second and third trimesters [60]. This structural adaptation is accompanied by metabolic reprogramming in the myocardium, including alterations in glycerophospholipid, nucleotide, and amino acid metabolism, as well as a reduction in glucose catabolism.

The brain undergoes progressive structural and functional changes during pregnancy. These include reductions in gray matter volume and cortical thickness, along with enhanced white matter microstructural integrity. These changes are closely linked to elevated levels of steroid hormones [61]. Hormonal fluctuations influence neuronal activity and neural circuit organization, contributing to behavioral adaptations such as increased appetite and reduced sensitivity to metabolic hormones, including leptin and insulin. These changes support the establishment of a positive energy balance [62].

The pituitary gland enlarges by approximately one-third during pregnancy, mainly due to increased estrogen levels. This hormonal influence leads to a proliferation of lactotrophs, which can account for up to 40% of the pituitary cell population by late gestation [63].

Thyroid physiology is also significantly altered during pregnancy. Total T4 and T3 levels increase due to elevated concentrations of thyroid-binding globulin, while thyroid-stimulating hormone (TSH) levels decline in response to the thyrotropic activity of placental human chorionic gonadotropin (hCG) [64]. Women with clinical or subclinical hypothyroidism during pregnancy are at heightened risk of developing dyslipidemia, which may contribute to a higher incidence of adverse pregnancy outcomes [65].

The endocrine pancreas adapts through hypertrophy of β-cells and expansion of the islets of Langerhans, thereby enhancing insulin production to meet increased metabolic demands [66]. In parallel, pregnancy is characterized by progressive insulin resistance, elevated circulating lipid levels, and increased adipose tissue accumulation [67]. Together, these adaptations ensure an adequate and sustained supply of nutrients to support fetal growth and development. [68]

**Figure 1:**
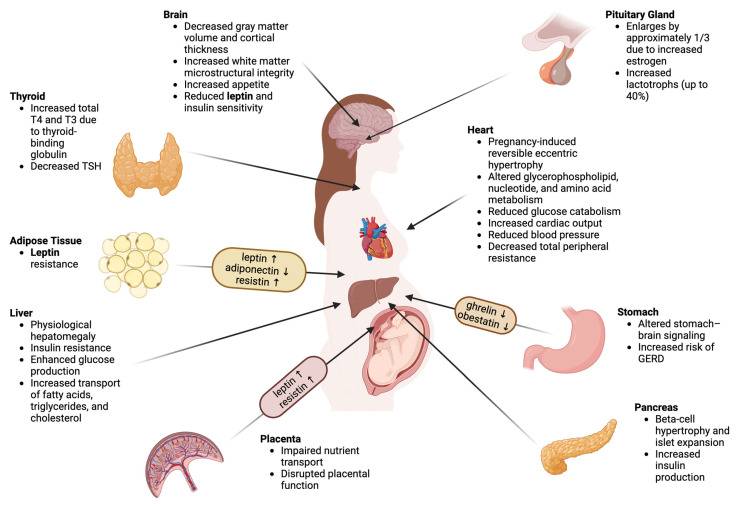
Organ-specific alterations in maternal overweight and obesity during pregnancy. The diagram summarizes metabolic and hormonal changes observed in key maternal organs and systems—including the brain, pituitary gland, thyroid, heart, liver, pancreas, adipose tissue, and placenta—in the context of overweight and obesity. The framed sections highlight the characteristic adipokine profile in obese women, with elevated leptin, resistin, and ghrelin, and reduced adiponectin and obestatin, which may impair maternal metabolic adaptations and contribute to pregnancy-related complications. ↑ indicates increased levels; ↓ indicates decreased levels.

Maternal overweight and obesity profoundly affect metabolic processes during pregnancy, influencing insulin sensitivity, appetite regulation, placental function, and adipokine secretion. The complex interaction of these metabolic and hormonal changes during pregnancy is illustrated in Figure 1, which summarizes adaptations across key maternal organs and tissues.

Excessive gestational weight gain, in addition to elevated pre-pregnancy BMI, is a major contributor to adverse maternal metabolic outcomes [69].

One of the most prevalent complications associated with maternal overweight and obesity is gestational diabetes mellitus. Excess maternal fat mass promotes insulin resistance, which, when combined with pregnancy-induced hormonal changes, substantially increases the likelihood of impaired glucose tolerance [70]. Women with obesity have a two- to four-fold higher risk of developing GDM compared with women of normal weight. GDM is associated with macrosomia, neonatal hypoglycemia, and an increased long-term risk of type 2 diabetes in both the mother and her child [5,71].

Overweight and obese pregnant women are at heightened risk of gestational hypertension and preeclampsia [72]. The pathophysiology is multifactorial and includes endothelial dysfunction, altered angiogenic signaling, systemic inflammation, and elevated oxidative stress. Preeclampsia in obese mothers not only contributes to maternal morbidity but also increases the risk of preterm delivery and intrauterine growth restriction [73,74].

Pregnancy itself is a hypercoagulable state, and obesity further amplifies this risk. Obese women have increased circulating pro-thrombotic factors, impaired fibrinolysis, and endothelial dysfunction, which predispose them to venous thromboembolism. Pulmonary embolism remains one of the leading causes of maternal mortality in this population [75,76].

Overweight and obesity are characterized by low-grade chronic inflammation and altered immune responses. During pregnancy, these changes increase susceptibility to urinary tract infections, wound infections (particularly after cesarean section), and postpartum mastitis. Such infections not only compromise maternal recovery but may also interfere with breastfeeding success and continuation [69,77,78,79].

Maternal obesity is associated with higher rates of cesarean section, labor induction, shoulder dystocia, and failed vaginal birth after cesarean. Technical difficulties in anesthesia and operative procedures further increase the risk of maternal morbidity. Moreover, excessive maternal adiposity is linked with abnormal uterine contractility and prolonged labor [80,81,82,83].

Lactation represents a unique physiological state in which maternal metabolism is extensively remodeled to prioritize milk synthesis. The energy cost of milk production increases maternal energy expenditure by approximately 500 kcal/day, requiring coordinated adjustments across multiple organs and endocrine systems [84]. Profound changes occur in multiple organs, including the liver, adipose tissue, skeletal muscle, the endocrine pancreas, and bones [85,86].

The liver reduces its own utilization of fatty acids and glucose, thereby redirecting these metabolites to the mammary gland, where they serve as essential precursors for lactose and milk lipids [87,88]. These hepatic adaptations are closely coordinated with hormonal changes, such as increased prolactin signaling and decreased progesterone activity, which collectively regulate the initiation and maintenance of lactogenesis [85,89].

Adipose tissue also undergoes profound remodeling. White adipose tissue becomes highly lipolytic, releasing free fatty acids and glycerol; the former serve as precursors for milk fat, whereas glycerol supports hepatic gluconeogenesis [90,91].

In skeletal muscles, substrate utilization decreases during lactation, sparing both glucose and fatty acids for the mammary gland. These adaptations are facilitated by altered insulin sensitivity and prolactin signaling, which regulate nutrient partitioning between maternal tissues and milk production [92,93].

The endocrine pancreas and systemic insulin sensitivity are also modified. Lactation is associated with improved insulin sensitivity, which supports the efficient transfer of glucose into mammary epithelial cells via GLUT1 transporters [94,95].

The skeleton contributes minerals—particularly calcium and phosphorus—to breast milk. This results in a temporary but reversible decline in bone mineral density, which typically recovers after weaning [86].

These organ-specific adaptations are tightly coordinated by hormonal changes. Prolactin stimulates milk synthesis and substrate uptake in the mammary gland, while the postpartum decline in progesterone is essential for the initiation of lactogenesis II (secretory activation). Insulin and cortisol further support substrate redistribution and mammary epithelial differentiation, whereas oxytocin regulates milk ejection and indirectly influences maternal energy balance by modulating stress responses [85,89]. Such metabolic remodeling not only ensures the nutritional adequacy of breast milk but also contributes to maternal energy balance and recovery in the postpartum period [91].

Obesity significantly affects lactation through hormonal, metabolic, and mechanical mechanisms. Women with obesity (BMI ≥30) are less likely to initiate breastfeeding, more prone to delayed onset of lactogenesis II, and tend to discontinue breastfeeding earlier. This is partly due to reduced prolactin responses to suckling and a smaller reduction in postpartum progesterone levels, both of which are essential for milk production. Excessive gestational weight gain (GWG), regardless of pre-pregnancy BMI, further decreases the likelihood of successful initiation and continuation of lactation [77,96]. Obesity is also associated with an altered endocrine environment in the mammary gland and a chronic low-grade inflammatory state, which may predispose women to inflammatory breast diseases, including mastitis—a complication that often leads to premature cessation of breastfeeding [77,97]. In addition, mechanical factors such as postpartum edema, nipple flattening, and difficulties with infant latch or positioning create further barriers to successful breastfeeding [98].

On the other hand, lactation exerts beneficial effects on maternal metabolic health by redistributing energy substrates and promoting fat mobilization, although these effects appear diminished in obese women [99]. Evidence shows that longer breastfeeding duration reduces the risk of substantial postpartum weight retention, particularly among women with normal pre-pregnancy BMI, whereas this benefit is less pronounced in overweight and obese mothers [100,101]. Moreover, mixed or exclusive formula feeding is associated with a significantly higher risk of postpartum weight retention compared to exclusive breastfeeding [102].

Collectively, these findings indicate that obesity not only impairs the initiation and maintenance of lactation but may also attenuate its potential metabolic benefits for the mother.

## 3. Leptin

Leptin is a hormone predominantly secreted by adipose tissue, circulating at concentrations proportional to body fat mass and responsive to acute changes in caloric intake. It is a key regulator of long-term energy balance, metabolism, and overall homeostasis [103]. High leptin levels indicate sufficient energy reserves, suppress appetite, and promote energy expenditure, whereas low levels signal energy deficiency, increasing hunger and reducing expenditure as an adaptive response. Leptin crosses the blood–brain barrier and binds to its long-form receptor (Ob-Rb; leptin receptor isoform b) in the hypothalamus, where it activates anorexigenic and inhibits orexigenic neurons to regulate appetite and maintain energy balance. Beyond the hypothalamus, leptin influences brain regions involved in salience, decision-making, and reward processing, and modulates functional connectivity between these regions [103,104,105,106,107]. It also acts as a critical link between adipose tissue and the reproductive system, signaling whether energy stores are sufficient for normal reproductive function, including puberty, ovulation, and pregnancy [108,109]. Chronically elevated leptin in obesity can lead to leptin resistance, reducing its regulatory effects on energy intake and metabolism [110].

Serum leptin levels are closely linked to body fat percentage and BMI in both non-pregnant adults and pregnant women. Individuals with elevated BMI exhibit higher serum leptin concentrations not only due to increased fat mass, but also because their adipocytes are larger. Moreover, leptin expression differs depending on adipose tissue type; omental adipocytes express lover levels of leptin mRNA compared with subcutaneous adipocytes [111,112,113].

Although adipose tissue is the primary source of leptin, during pregnancy the placenta also contributes to its production and releases it predominantly into the maternal circulation, with a smaller proportion reaching the fetal circulation [110,114,115]. Serum leptin concentrations in pregnant women are approximately twofold higher than in non-pregnant women [116]. The presence of leptin in the placenta has been observed as early as the 7th week of pregnancy [117]. Leptin levels increase by 30% by the 12th week of pregnancy, remain relatively stable in mid-pregnancy, and return to pre-pregnancy levels within 24 h after delivery [118]. Since maternal serum leptin levels drop sharply postpartum, it is likely that the observed increase during pregnancy is primarily due to placental leptin release rather than adipose tissue production [119].

Leptin is both synthesized by the mammary gland and transferred from maternal circulation into breast milk. It is present in milk as part of the fat globule fraction, which accounts for the higher leptin concentrations typically observed in whole milk compared with skimmed milk [47,120,121,122].

Its levels peak in colostrum and progressively decline throughout lactation, with significantly higher concentrations observed in maternal serum than in breast milk [37,107,123]. In a cohort of lactating women, colostrum leptin concentrations averaged 3.28 ± 0.41 ng/mL, showing a strong inverse correlation with the duration of lactation over the first 180 days postpartum. Leptin levels in both colostrum and mature milk correlated positively with maternal serum leptin and, in mature milk, were also significantly associated with maternal cortisol, prolactin, and thyroxine concentrations [123,124]. Notably, leptin concentrations in breast milk did not differ between term and preterm births [124].

### 3.1. Pregnancy

The study by Vinod K. Misra and Sheri Trudeau [29] was the first to demonstrate that women with overweight or obesity (pre-pregnancy BMI >26 kg/m^2^) have a distinct leptin profile throughout pregnancy compared with normal weight women. In a prospective cohort of 143 singleton pregnancies, serum leptin levels were measured at 6–10, 10–14, 16–20, 22–26, and 32–36 weeks of gestation. Overweight/obese women had significantly higher leptin levels per unit of body weight throughout pregnancy. While this ratio increased in normal-weight women, it significantly declined in overweight/obese women, suggesting that they produce progressively lower amounts of leptin per unit of adipose or placental tissue as pregnancy progresses.

Pregnant women with overweight and obesity exhibited significantly greater total and subcutaneous abdominal fat compared to those with a normal BMI, while no differences were observed in visceral fat. Leptin levels were markedly higher (66.3 ± 34.2 vs. 35.7 ± 19.3 ng/mL) and showed a strong correlation with total fat mass [33].

Serum leptin levels were also examined to investigate the relationship between leptin (measured between the 9th and 26th week of pregnancy) and preeclampsia in a cohort of 430 women with preeclampsia and 316 normotensive primigravidas. The study found that an increase in BMI was significantly associated with higher leptin levels, independent of factors such as gestational age at blood collection, maternal age, smoking status, and socioeconomic factors [125]. A multiple linear regression analysis by Maple-Brown et al. [31] found that pre-pregnancy BMI was the strongest independent determinant of leptin levels. When pregnancies were stratified into high leptin (>20 ng/mL) and low leptin (<20 ng/mL) groups, women in the high-leptin group were older and had higher pre-pregnancy BMI and body weight at the end of pregnancy compared to those in the low-leptin group [126].

A study by Branham et al. [127] found a weak correlation between serum leptin levels at 24–28 weeks of gestation and BMI in the first trimester. It is suggested that leptin levels may have been influenced by the glucose challenge test, as insulin regulates leptin production.

Research conducted at the Medical University of Łódź demonstrated a positive correlation between leptin levels and BMI in obese pregnant women at 20–24 weeks of gestation [34]. Furthermore, women between 24 and 34 weeks of gestation who were overweight or obese had significantly higher concentrations of leptin (43.44 ± 31.41 vs. 21.29 ± 12.67 ng/mL), and C-peptide (2.77 ± 1.88 vs. 2.25 ± 1.42 ng/mL) compared with lean women. In this cohort, a significant correlation was observed between leptin and C-peptide levels [32].

Despite these findings, overweight and obese women remain at risk for excessive GWG [128,129]. A study conducted on healthy women who delivered by cesarean section found that those with excessive weight gain had higher leptin levels, and their newborns exhibited elevated LDL-C levels [130]. Logan et al. [131] reported that early pregnancy weight gain was associated with higher maternal serum leptin levels in the postpartum period.

A prospective cohort study involving 675 women revealed that higher leptin levels in the second trimester were linked to greater subsequent gestational weight gain, independent of maternal obesity and other factors. This finding contrasts with the expected physiological regulation of leptin in non-pregnant individuals, with the strongest association observed in women classified as overweight at the beginning of pregnancy [132]. Leptin may regulate weight gain differently in the first and second halves of pregnancy. In the second trimester, higher leptin levels are associated with greater weight gain, especially in women with overweight, suggesting that leptin may stimulate a positive energy balance in the latter half of pregnancy. In the first trimester, higher leptin levels were linked to lower weight gain, but this association disappeared after adjusting for body fat percentage [132].

In another study, Malti et al. [35] found that leptin levels were over 40% higher and oxidative stress markers were more than 25% higher in obese mothers compared with controls across all trimesters. ANOVA analysis revealed significant effects of both obesity and gestational stage on leptin and oxidative stress markers. Notably, leptin levels increased from early to late pregnancy in both groups but were more strongly influenced by obesity than by gestational duration.

### 3.2. Lactation

Several studies have demonstrated a strong positive correlation between milk leptin concentration and maternal adiposity indicators. In two independent cohorts, milk leptin levels showed significant associations with maternal body weight, BMI, fat mass, and body fat percentage [133,134]. Moreover, significant correlations have also been observed with pre-pregnancy BMI and maternal anthropometric measures during pregnancy, including at the 15th and 32nd gestational weeks [135].

Although circulating serum leptin may contribute to leptin levels in milk, the correlation between serum leptin and milk leptin was consistently weaker than the correlation between maternal adiposity and milk leptin. This suggests that the mammary gland may selectively concentrate leptin in a manner that reflects maternal fat stores more directly than circulating hormone levels alone. Importantly, no such association was observed for other appetite-regulating hormones [134].

In the study by Kugananthan et al. [36], higher maternal fat mass percentage was significantly associated with increased leptin concentrations in both whole and skimmed breast milk, indicating that maternal adiposity influences leptin content regardless of milk fraction.

Notably, leptin concentrations remained stable throughout the first year of lactation, suggesting that maternal body composition plays a more prominent role than the stage of lactation in determining milk leptin levels [36].

Recent findings from the BLOOM study further support the strong relationship between maternal adiposity and leptin levels in both serum and breast milk. In this cohort, overweight and obese mothers presented with significantly elevated leptin levels compared with normal weight women—with obese mothers exhibiting up to 6.2-fold higher breast milk leptin concentrations. A particularly strong positive correlation was observed between maternal fat mass percentage and both serum and milk leptin levels [30].

Interestingly, the study also identified associations between serum leptin and cardiometabolic health markers, suggesting that leptin may reflect underlying metabolic risk. Moreover, higher adherence to a Mediterranean-style diet was associated with lower leptin concentrations in breast milk among OW/OB mothers, even after adjusting for adiposity, indicating a potential protective role of healthy dietary patterns in modulating milk composition [30].

Pre-pregnancy BMI was found to be positively associated with breast milk leptin concentrations in both crude and adjusted models. Additionally, excessive GWG was independently linked to higher leptin levels in breast milk, while greater postpartum weight loss showed a significant negative association with milk leptin concentrations, independent of maternal BMI [37].

Additionally, the study by Fields et al. [39] observed a 33.7% reduction in breast milk leptin concentration from month 1 to month 6 postpartum, likely reflecting the gradual reduction in maternal fat mass after delivery.

These results differ from those of Zamanillo et al. [38], who reported that leptin concentrations in breast milk declined over the course of lactation in normal weight mothers but remained stable in overweight/obese women, who exhibited 2.8-fold higher leptin levels at one month postpartum compared with their normal weight counterparts.

The association between maternal adiposity and breast milk leptin concentration has been confirmed in numerous studies, including those by Fields et al. [39] (overweight and obese mothers had 96.5% and 315.1% higher leptin levels than normal weight mothers, respectively), Savino et al. [136], Chan et al. [48], Enstad et al. [121], Schneider-Worthington et al. [122], Young et al. [137] and Sims et al. [138]. Interestingly, Logan et al. [139] were the first to report a non-linear relationship between BMI and breast milk leptin.

These findings emphasize the complex interplay between maternal metabolic status, dietary habits, and the hormonal composition of breast milk, with potential long-term implications for offspring development.

## 4. Adiponectin

Adiponectin is a pleiotropic adipokine with both anti-inflammatory and pro-inflammatory actions. It exerts its biological effects through two receptors, AdipoR1 and AdipoR2, which mediate a broad spectrum of metabolic and reproductive functions [140,141,142,143]. Metabolically, adiponectin stimulates fatty acid oxidation in skeletal muscle, inhibits hepatic glucose production, enhances insulin sensitivity, promotes apoptosis in cancer cells, and exhibits antioxidant properties, thereby contributing to whole-body energy homeostasis [141,144].

Beyond metabolism, adiponectin plays an important role in reproduction. It modulates key reproductive hormones, including kisspeptin, GnRH, and gonadotropins, linking energy status with reproductive function. In females, adiponectin regulates steroidogenesis in granulosa and theca cells, contributes to oocyte maturation and embryo development, and, through its presence in placental and endometrial cells, is thought to influence embryo implantation, trophoblast invasion, and fetal growth [145].

Adipose tissue is the primary source of circulating adiponectin during pregnancy, and its levels fluctuate significantly depending on maternal body mass [146]. Both total adiponectin and its high-molecular-weight (HMW) form decrease in obesity and increase following weight loss [147,148,149]. Reduced serum adiponectin concentrations have been linked to obesity, type 2 diabetes, dyslipidemia, and cardiovascular disease [20]. Recent studies show that adiponectin is predominantly secreted during the daytime, following a circadian rhythm. Although the influence of circadian regulation on adiponectin expression is not yet fully understood, several metabolic disorders—including obesity and insulin resistance—have been associated with circadian rhythm disturbances [150].

While adiponectin levels are naturally lower during pregnancy, studies suggest that this difference disappears after adjusting for pregnancy-related increases in adipose tissue and insulin resistance [151]. The concentration of adiponectin, particularly the HMW form, reaches its lowest point in the third trimester, coinciding with peak maternal insulin resistance [152].

Adiponectin is present in breast milk, with an average concentration of approximately 19 ng/mL (range 4.2–87.9 ng/mL). It has been reported to be more abundant in cord blood (30.6 mg/L) than in breast milk (10.9 ng/mL) or maternal serum (8.6 mg/L) [20]. Moreover, adiponectin concentrations in breast milk are positively correlated with maternal serum levels [153]. Longitudinal studies have shown that adiponectin levels decrease as lactation progresses, with significantly lower concentrations in both maternal serum and breast milk at 4 months compared with 1 month postpartum, and slightly lower levels at 4 months than at 6 weeks postpartum [135,154].

### 4.1. Pregnancy

Research by Suto et al. [42] found that adiponectin levels were significantly lower in overweight and obese women as early as the first and second trimesters of pregnancy.

Cross-sectional studies have demonstrated a negative correlation between adiponectin levels and gestational stage, particularly in women with normal body weight. However, overweight patients consistently exhibited lower adiponectin levels from the beginning of pregnancy [41]. Jansson et al. [155] also confirmed a negative correlation between BMI and adiponectin levels in the first trimester, with high maternal fat intake being inversely correlated with this hormone. Also, the study by Ozias et al. [33] demonstrated no significant association between adiponectin and body composition.

Adiponectin plays a crucial role as an indicator of maternal metabolic health and a predictor of preeclampsia, especially when factors such as BMI, age, parity, and family history of diabetes are considered [156]. Obese women tend to have significantly lower adiponectin levels compared with those with normal body weight, which may lead to increased placental nutrient transport and contribute to fetal overgrowth [115]. Maternal hypoadiponectinemia can have functional consequences, impairing biological signaling in various tissues, including the placenta [157].

The importance of adiponectin as a marker of preeclampsia has been further confirmed by studies showing that overweight and obese women with severe preeclampsia had significantly lower adiponectin levels compared to those with normal body weight (8.4 ± 5.3 vs. 12.6 ± 6.0 ng/mL) [111]. Beyond BMI, race also appears to influence the leptin-to-adiponectin ratio (LAR) during pregnancy—research indicates that Black women with obesity exhibited a higher LAR early in pregnancy, which continued to rise as pregnancy progressed [158].

### 4.2. Lactation

Breast milk adiponectin concentrations increased significantly after feeding in both normal-weight and obese mothers. However, post-feed adiponectin levels were significantly lower in mothers with obesity (12.84 ± 2.33 ng/mL) compared with their normal-weight counterparts (13.95 ± 0.25 ng/mL), indicating that maternal adiposity may negatively influence adiponectin secretion into milk, particularly in the postprandial phase [43].

Additionally, according to longitudinal data, higher maternal postpartum BMI was significantly associated with elevated adiponectin concentrations in breast milk [46]. Similarly, Clark et al. [44] and Yu X et al. [45] reported that the concentrations of adipokines in the milk of mothers with obesity were higher than in those with normal body weight.

In contrast, the study by Christensen et al. [47] showed that breast milk adiponectin concentrations were not associated with maternal BMI. Although its levels declined during early lactation (up to approximately 3.5–6 months), they remained stable thereafter, suggesting that adiponectin regulation in breast milk may be independent of maternal adiposity. Moreover, a similar pattern was observed by Zamanillo et al. [38], who reported a ≈20% decrease in adiponectin over time exclusively in normal-weight mothers, with no such change among overweight or obese women.

Similar conclusions were reached by other authors, including Chan et al. [48] and Sadr Dadres et al. [37] who also reported no significant association between maternal BMI and breast milk adiponectin levels.

## 5. Ghrelin

Ghrelin, often referred to as the “hunger hormone,” is a key orexigenic peptide with pleiotropic functions [159]. It is predominantly produced by endocrine P/D1 cells in the gastric fundus, with smaller amounts secreted by the hypothalamus, kidneys, heart, pancreatic cells, and placenta [160]. Synthesized as preproghrelin, it undergoes processing to proghrelin and further cleavage into the mature 28-amino-acid peptide. Its biological activity requires acylation of serine-3 by ghrelin O-acyltransferase, which enables binding to the growth hormone (GH) secretagogue receptor type 1α (GHS-R1α) [161,162,163].

Ghrelin stimulates appetite and food intake by acting primarily on the arcuate nucleus of the hypothalamus. Its secretion rises during fasting and weight loss, whereas it falls under conditions of positive energy balance, such as after food intake and in obesity [20].

Its hallmark functions include stimulation of GH release, promotion of food intake, fat deposition, and regulation of glucose homeostasis. Ghrelin exerts metabolic effects by inhibiting insulin secretion, modulating gluconeogenesis and glycogenolysis, and decreasing thermogenesis to regulate energy expenditure [164,165].

Beyond energy balance, ghrelin also acts as an endocrine factor in stress homeostasis [166]. Importantly, ghrelin exists in two major forms: acylated (active) ghrelin, required for binding to GHS-R1α, and desacyl ghrelin (also known as unacylated ghrelin), which, influences cell proliferation and adipogenesis and counteracts several metabolic actions of the active form [167,168].

Plasma ghrelin levels are inversely correlated with BMI, being elevated in catabolic states such as anorexia nervosa or cachexia and reduced in obesity [169]. A recent meta-analysis demonstrated that individuals with obesity—regardless of sex—had significantly lower circulating levels of both total and active ghrelin compared with normal weight subjects. Total ghrelin was reduced by 145.53 pg/mL, and active ghrelin by 53.22 pg/mL [170].

During pregnancy, ghrelin levels peak in the second trimester and subsequently decline as pituitary GH is gradually replaced by placental GH [171,172]. Serum ghrelin concentrations reach their maximum at the 18th week (1200 ± 90 pg/mL) and were at their lowest by the end of the third trimester (870 ± 60 pg/mL), representing an average decline of 27.7% from the peak values [172].

Dündar et al. [173] reported that free ghrelin levels in breast milk (1280 ± 32.6 pg/mL) were markedly higher than in maternal serum (246 ± 65.8 pg/mL) and cord blood (346 ± 120 pg/mL). In contrast, total ghrelin concentrations were greatest in cord blood, followed by breast milk, and lowest in maternal serum. Moreover, concentrations were higher in whole milk than in skimmed milk [20]. Longitudinal analyses further demonstrated that active ghrelin in breast milk is lowest in the early postpartum period (450 ± 25 pg/mL), increasing to 801 ± 43 pg/mL by 180 days, whereas total ghrelin rises from 880 ± 80 pg/mL to approximately 3250 ± 380 pg/mL at 91–180 days postpartum. During the same period, serum total ghrelin concentrations in breastfeeding women increased, whereas serum active ghrelin levels declined significantly [167]. In comparative analyses, ghrelin concentrations in colostrum (70.3 ± 18 pg/mL), transitional milk (83.8 ± 18 pg/mL), and mature milk (97.3 ± 13 pg/mL) were lower than those measured in the corresponding maternal plasma samples (95 ± 16 pg/mL at day 1; 111 ± 13 pg/mL at day 10; 135 ± 16 pg/mL at day 15). Interestingly, plasma ghrelin concentrations were lower in lactating than in non-lactating women [50].

### 5.1. Pregnancy

Tehranian et al. [49] found no significant differences in plasma ghrelin levels between pregnant women with overweight and those with a normal BMI from the first to the second trimester. Additionally, no correlation was observed between ghrelin levels and gestational weight gain in either group.

A study conducted on non-diabetic pregnant women with a pre-pregnancy body mass index of 25.0–34.9 kg/m^2^, assessed between the 28th and 32nd weeks of gestation, identified a positive association between maternal stress and ghrelin levels in women with overweight and obesity. These findings suggest a potential link between psychological factors and the regulation of appetite-related hormones during pregnancy [174]. In addition, elevated fasting serum acylated ghrelin concentrations in the second trimester have been linked to greater gestational weight gain [175].

### 5.2. Lactation

The concentration of ghrelin in pre-feed breast milk was significantly higher in mothers with obesity compared with those with normal weight, indicating that maternal adiposity influences ghrelin levels independently of feeding stage. Notably, although ghrelin levels decreased over the course of lactation in both groups, they remained consistently elevated in obese mothers [43]. Additionally, weak correlations between BMI and ghrelin levels in both plasma and milk were observed in a small cohort, with ghrelin concentrations increasing as maternal weight decreased postpartum [50]. On the other hand, according to Yu and colleagues [45], maternal BMI was also found to be inversely associated with ghrelin concentrations in breast milk, indicating that higher maternal BMI is linked to reduced ghrelin levels in breast milk.

In contrast, in the study by Andreas et al. [51] breast milk ghrelin measured at 1 week and 3 months postpartum showed no correlation with maternal BMI assessed at the time of milk collection.

## 6. Obestatin

Obestatin is a peptide derived from the same preproghrelin precursor as ghrelin, but it exerts largely opposite biological effects [176,177]. Initially identified as an anorexigenic hormone, obestatin has been implicated in the regulation of appetite and energy homeostasis, gastrointestinal motility, lipid metabolism, and glucose balance. Beyond its metabolic actions, obestatin also influences cell proliferation, apoptosis and exerts cardioprotective and anti-inflammatory effects, suggesting a pleiotropic role in both physiology and disease [177,178,179].

The concentrations of obestatin are significantly lower in obese women, including those with diabetes [178,180]. In fact, levels in the normal-weight group were on average 64.19 pg/mL higher than in the obese group [170].

Moreover, in patients with anorexia nervosa or obesity, circulating obestatin levels have been shown to correlate negatively with BMI, leptin, insulin, glucose, and the homeostasis model assessment of insulin resistance (HOMA-IR), while displaying positive correlations with both acyl-ghrelin and desacyl-ghrelin. These findings suggest that the basal secretion of obestatin and ghrelin may be co-regulated, with both hormones being influenced by adiposity and insulin resistance [177].

Obestatin concentrations in breast milk have been reported to exceed those in maternal circulation. In one study, obestatin levels in colostrum (538.9 ng/L) and mature milk (528.5 ng/L) were more than twice as high as the corresponding serum concentrations (270.3 and 289.4 ng/L, respectively) [176]. Another report noted colostrum obestatin concentrations of 290 ± 160 ng/L [181]. Similarly, Savino et al. [182] found mean serum obestatin levels of 759.1 ng/L in lactating mothers, while breast milk contained comparable concentrations (846.6 ng/L), with a positive correlation between maternal and infant serum obestatin levels.

### 6.1. Pregnancy

To date, no studies have specifically evaluated the association between maternal BMI and circulating obestatin levels during pregnancy. However, elevated serum obestatin concentrations have been reported in women with preeclampsia, suggesting a potential role of this peptide in pregnancy complications independent of maternal adiposity. The study by Wu et al. [183] demonstrated a decrease in maternal serum ghrelin concentrations together with a significant increase in obestatin levels in women with preeclampsia compared with those with normal pregnancies. In blood samples collected at 20–38 weeks of gestation, maternal serum obestatin concentrations were 276.35 ± 15.38 ng/L in the preeclampsia group and 223.53 ± 18.61 ng/L in the control group, with the difference reaching statistical significance.

### 6.2. Lactation

Evidence during lactation is also limited; one study reported lower obestatin concentrations in breast milk at 3–7, 14–15, and 30 days postpartum among mothers with a higher body fat percentage [52], but data directly linking maternal BMI to milk or serum obestatin remain lacking.

## 7. Resistin

Resistin is a cysteine-rich peptide hormone and pro-inflammatory adipokine, produced predominantly by macrophages in humans and expressed in various tissues, including the placenta [184,185]. Notably, resistin expression is greater in intra-abdominal than in subcutaneous fat depots [186]. Resistin reduces the insulin sensitivity of target tissues—such as skeletal muscle, adipose tissue, and liver—by impairing glucose transporter protein type-4 (GLUT4) translocation and disrupting glucogenic metabolism. In the liver, it influences lipid metabolism by decreasing AMP-activated protein kinase phosphorylation, thereby reducing β-oxidation and increasing esterified fatty acids and triacylglycerides, leading to lipid accumulation in the hepatic parenchyma [187]. Insulin resistance is strongly associated with central obesity [186]. These observations have led to the hypothesis that resistin serves as a molecular link between obesity and type 2 diabetes, potentially acting at one or more steps in the insulin-signaling pathway to induce insulin resistance [188].

Beyond its metabolic effects, resistin also plays an important role in immune and inflammatory responses. It stimulates the production of pro-inflammatory cytokines, including TNF-α, IL-6, and IL-1β, and exhibits potent pro-inflammatory activity by strongly upregulating IL-6 and TNF-α expression, responding to TNF-α challenge, and enhancing its own activity via a positive feedback loop [189,190]. Plasma resistin concentrations show no significant correlation with body mass index, waist circumference, HOMA-IR, fasting glucose, or fasting insulin levels. In contrast, a weak but statistically significant positive association has been reported with abdominal subcutaneous fat [191].

During pregnancy, resistin is thought to contribute to the physiological insulin resistance that develops as gestation progresses, thereby ensuring an adequate nutrient supply for the growing fetus [33]. Already in the first trimester, circulating resistin levels are slightly higher than in non-pregnant women—for example, median values of 12.8 ng/mL (range: 4.6–81.3) have been reported, compared with 10.4 ng/mL (6.5–11.75) in controls [192]. The study by Chen et al. [193], however, found no significant differences between non-pregnant women and those in the first or second trimesters. Despite these discrepancies, a consistent pattern emerges in the third trimester, when resistin concentrations rise significantly [175,194].

Interestingly, resistin concentrations in umbilical cord (UC) blood are often higher than in maternal plasma, and maternal and UC levels are positively correlated. Both maternal and fetal concentrations also demonstrate a positive correlation with gestational age, suggesting a coordinated regulation or possible transfer during pregnancy [195].

Following delivery, resistin is present in breast milk, peaking at ~1.71 ng/mL in the first 1–3 days postpartum alongside high maternal serum levels (~5.8 ng/mL). Both decline over the following months to ~0.67 ng/mL in milk and ~2.06 ng/mL in serum by 3–6 months. The correlation between serum and milk resistin concentrations is strong, and both are positively associated with maternal hormonal status, including estradiol, progesterone, prolactin, thyroxine, triiodothyronine, cortisol, leptin, and inflammatory markers such as C-reactive protein [123].

### 7.1. Pregnancy

Previous research has yielded inconsistent findings regarding maternal resistin levels during pregnancy. One study found no significant associations between resistin concentrations and maternal outcomes, such as pre-pregnancy BMI or gestational weight gain [40]. In contrast, another investigation reported that the median maternal resistin concentration was higher among obese women compared with those with normal body weight (1.41 [1.02–1.95] ng/mL vs. 1.31 [0.74–1.52] ng/mL) [53]. Furthermore, the study by Ozias et al. [33] demonstrated a significant association between resistin and visceral fat in pregnant women.

### 7.2. Lactation

According to Andreas et al. [51], no association was observed between breast milk resistin concentrations and maternal BMI at 1 week or 3 months postpartum, with BMI assessed at the time of milk sampling. Similarly, Santosa et al. [54] found no correlation between breast milk resistin concentrations obtained at 1 month postpartum and maternal anthropometric parameters, including pre-pregnancy body weight and BMI, body weight at delivery, and body weight and BMI at 1 month postpartum.

## 8. Discussion

**Figure 2:**
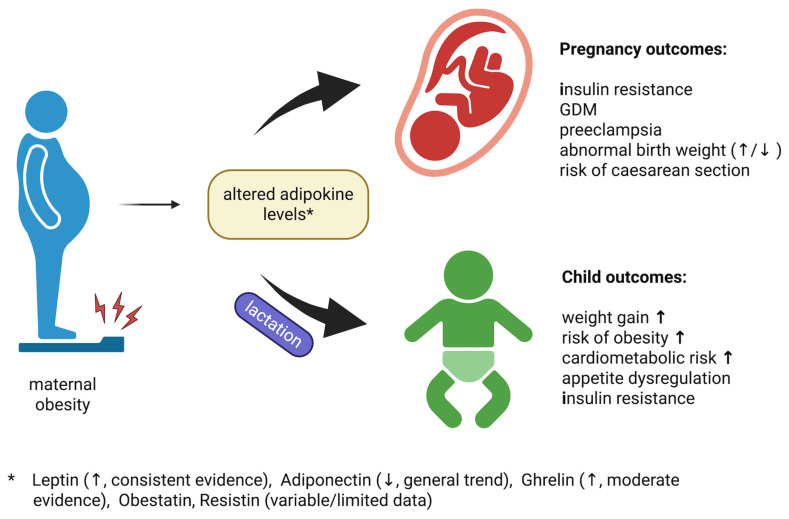
Impact of maternal obesity–related adipokine alterations on pregnancy complications and offspring metabolic outcomes. ↑ indicates an increase; ↓ indicates a decrease.

Maternal obesity is associated with a wide range of alterations during pregnancy and lactation, affecting metabolic adaptations, placental function, and breast milk composition [1,5,196]. Among these, changes in adipokine profiles serve as key modulators of maternal health and offspring development. Figure 2 provides an overview of these relationships, illustrating how disturbances in adipokine levels contribute to gestational complications and shape long-term metabolic programming in children [197].

Obesity is a significant risk factor for preeclampsia, and elevated leptin levels proportional to adipose tissue mass may contribute to its pathogenesis through inflammatory signaling and endothelial dysfunction [198]. Maternal obesity is associated with reduced adiponectin and elevated leptin and visfatin concentrations both in mid-pregnancy (18–22 weeks of amenorrhea) and at the time of delivery [199]. Current evidence suggests that preeclampsia is associated with elevated levels of leptin, chemerin and fatty acid-binding protein 4 (FABP4) across all trimesters of pregnancy and clinical subtypes of the disorder. However, data regarding the role of other adipokines remain inconsistent [200]. Leptin levels correlate positively with BMI and negatively with birth weight. Both high BMI (≥28) and leptin were independent risk factors for preeclampsia, with leptin mediating 22.5% of BMI’s effect [201]. It is also suggested that plasma leptin levels appear to rise prior to the onset of clinical symptoms of preeclampsia [202].

Lifestyle during pregnancy influences maternal health, but the impact of physical activity on leptin, resistin, and related factors remains unclear. A study analyzed data from two lifestyle intervention programs that assessed the effect of exercise (twice a week, 60–90 min per session) on adipokines during pregnancy. The results showed a positive correlation between fat mass at 14 weeks of pregnancy and both leptin and resistin levels at subsequent time points. At 36 weeks, leptin levels were significantly higher in the control group, with no differences for resistin [203]. Moreover, exercise during pregnancy has been shown to increase apelin levels, which may alleviate reduce preeclampsia symptoms by enhancing endothelial nitric oxide synthase, nitric oxide, placental growth factor, and vascular endothelial growth factor, while lowering soluble fms-like tyrosine kinase, soluble endoglin, and oxidative stress [204]. These findings highlight the importance of understanding how physical activity and fat mass influence maternal leptin, resistin and apelin levels during pregnancy. Future studies should investigate whether interventions that target maternal adipokine profiles through dietary or lifestyle modifications can positively influence fetal metabolic programming and reduce the long-term risk of metabolic diseases.

The adipokine profile in obese pregnancies is not merely an indicator of metabolic status, but also an active contributor to the development of pregnancy-related pathologies. Adipokines may serve as predictors of future metabolic risk in women after pregnancy and could also offer novel therapeutic targets for managing insulin resistance and impaired glucose tolerance during gestation, thereby helping to prevent various complications associated with GDM [12]. In a prospective longitudinal study, maternal levels of several adipokines measured early and mid-pregnancy were significantly associated with the risk of GDM, with FABP4, chemerin, IL-6, and leptin linked to increased risk, while higher soluble leptin receptor and adiponectin levels were associated with reduced risk. These associations remained statistically significant even after adjusting for pre-pregnancy BMI, although their strength was attenuated, indicating that maternal adiposity partially mediates the relationship between adipokines and GDM risk [205].

In addition to the five adipokines discussed above, other biomarkers and predictive approaches are being explored to better understand the metabolic adaptations in pregnancy complicated by overweight and obesity. For example, a study by Mario Solis-Paredes et al. [206] developed artificial neural network models to predict biomarker concentrations in late pregnancy. These models demonstrated high accuracy, achieving regression coefficients above R^2^ = 0.945. Pre-pregnancy BMI was the key variable for predicting adiponectin and carbonylated proteins (37%), while gestational age played the most significant role in forecasting resistin and malondialdehyde levels (34%).

Moreover, novel adipokines are emerging as potential modulators of maternal metabolism. Adipolin, for instance, has been implicated in obesity, insulin resistance, and glucose metabolism, and has been shown to improve insulin and glucose tolerance in diet-induced obese mice [207]. However, no significant differences were found in adipolin levels between pregnant women with gestational diabetes, those with overweight/obesity without gestational diabetes, and the control group, nor between the overweight and obesity groups [208].

While pregnancy represents a period of profound hormonal and metabolic change, the influence of maternal adipokines does not end with delivery. These bioactive molecules are transferred to the newborn via breast milk, where they may continue to shape metabolic programming, growth trajectories, and long-term health outcomes [3,5,21,22].

The impact of breast milk composition on infant growth and body composition appears to vary according to maternal BMI [209]. Infants born to overweight or obese mothers tend to exhibit accelerated growth and excessive weight gain during the first year of life, even when exclusively breastfed. This early rapid growth has been identified as a potential risk factor for developing obesity later in life [210]. One proposed explanation is that infants of these mothers may consume greater volumes of breast milk, as suggested by observations of increased feeding eagerness and subsequent weight gain in this population [43].

Women with elevated BMI are also more likely experience difficulties in maintaining lactation, with increased rates of early breastfeeding cessation [211]. Moreover, the higher incidence of cesarean delivery among overweight and obese mothers may further compromise successful initiation of breastfeeding, as supported by meta-analytic evidence linking cesarean section with lower rates of early breastfeeding initiation [212].

Interestingly, lower concentrations of insulin, leptin, and adiponectin have been observed in the milk of mothers who exclusively breastfeed, compared with those who introduce formula or complementary foods. This may reflect either a dilution effect due to higher milk volumes or be associated with greater postpartum weight loss typically observed in exclusive breastfeeding [48].

A short-term reduction in maternal energy, sugar, and fat intake has been shown to significantly decrease the concentrations of leptin, insulin, and adiponectin in breast milk, without affecting its macronutrient composition. These changes were accompanied by a modest reduction in maternal weight and fat mass, suggesting that alterations in maternal metabolic status may rapidly influence the hormonal profile of breast milk; most importantly; notably, no effects were observed on 24-h milk production or infant growth [213].

Among milk-transferred hormones, adiponectin has received considerable research attention, although findings remain inconsistent. These discrepancies may be due to the fact that breast milk adiponectin is more closely related to pre-pregnancy BMI rather than current BMI [122]. Interestingly, one of the studies found a positive association between maternal serum adiponectin levels and infant daily weight gain, whereas no such relationship was observed for adiponectin concentrations in breast milk [214]. While the role of adiponectin in metabolic regulation and early-life programming remains incompletely understood, some studies have suggested that higher adiponectin levels in breast milk may be linked to increased adiposity in children [38]. It has been hypothesized that milk-derived adiponectin plays a critical role during the transition to complementary feeding in infancy by promoting catabolic pathways, thereby helping to reduce the risk of overweight and obesity later in life [210].

Another adipokine that has been extensively studied in both pregnancy and lactation is leptin, due to its dual role in maternal metabolic adaptation and fetal programming. Maternal serum leptin levels rise in late pregnancy and subsequently decrease after delivery—a pattern often referred to as the “leptin surge”—which is thought to play a key role in the development of hypothalamic appetite-regulating pathways in the fetus [37].

Experimental data further support the role of leptin in fetal growth and metabolic programming. Cord blood leptin concentrations are thought to reflect intrauterine nutritional status and fetal fat stores, with lower levels typically observed in small-for-gestational-age (SGA) neonates and higher levels in large-for-gestational-age neonates, as compared with appropriate-for-gestational-age infants [215,216].

Furthermore, cord blood leptin has been shown to predict postnatal growth trajectories: lower levels are associated with accelerated weight gain during infancy, while higher levels are linked to slower weight gain over the first two years of life, independent of birth weight [115,217,218]. However, in later childhood and adolescence, higher cord blood leptin levels appear to be linked with excess adiposity. In the HAPO Follow-Up Study, higher cord leptin was associated with greater childhood adiposity, even after adjustment for maternal BMI and glucose, and remained significantly related to body-fat percentage, fat mass, sum of skinfolds, and increased odds of overweight and obesity [216]. In the UK cohort, higher cord leptin predicted slightly higher BMI and waist circumference at 9 years, but associations attenuated to null by 17 years, highlighting heterogeneity over time [219]. These findings suggest that leptin exerts biological effects in early life and that infants may remain responsive to leptin despite relatively high circulating levels, potentially as an adaptive response to intrauterine nutritional or metabolic stress. Taken together, cord blood leptin may serve as a potential biomarker of fetal fat stores and short-term growth patterns but appears to have limited utility for predicting long-term obesity.

Accordingly, breastfed infants of obese mothers are exposed to higher concentrations of leptin in milk [138]. However, this does not appear to provide a protective effect against excessive weight gain in these children. On the contrary, they seem to be at greater risk of developing obesity—possibly due to impaired leptin signaling or resistance, which diminishes the expected appetite-regulating function of milk leptin [38,220]. Leptin primarily acts on the hypothalamus to regulate energy balance by suppressing appetite. Yet, chronically elevated levels—as observed in obesity—may lead to leptin resistance, a state in which the body no longer responds effectively to leptin’s anorexigenic signals. This resistance can result in increased appetite and subsequent weight gain, potentially explaining the paradoxical link between high milk leptin exposure and greater adiposity in offspring [221]. Specifically, studies have shown that elevated milk leptin levels are linked to reduced weight-for-length (WFL) z-scores at 4 months and 1 year of age, as well as lower total and trunk fat mass at 6 months. This suggests that leptin may exert a protective effect against excessive fat gain in early life, although this effect might be weaker in infants of obese mothers due to reduced leptin sensitivity [39,48,221]. Furthermore, emerging evidence indicates that leptin in breast milk may influence the infant gut microbiota, promoting beneficial microbial metabolic pathways and reducing intestinal inflammation [222].

Despite the well-recognized benefits of breastfeeding, concerns persist that maternal overweight or obesity during pregnancy and lactation may contribute to offspring adiposity and increase the risk of later overweight/obesity. Indeed, children of mothers with pre-pregnancy overweight or obesity have a substantially higher risk of overweight/obesity throughout childhood, with risk rising progressively across maternal BMI classes [21,223]. Nevertheless, available evidence indicates that breastfeeding remains strongly advisable in this population. In a multi-cohort analysis of 8134 mother-child dyads, breast milk exposure—regardless of maternal BMI category—was associated with a lower child BMI z-score between ages 2 and 6 years [224]. Similarly, six months of exclusive breastfeeding (vs. none) was linked to approximately 60% lower odds of obesity, with an inverse gradient for body fat percentage across breastfeeding duration; these associations persisted after adjustment for maternal BMI [225]. Notably, an additive interaction has been observed whereby the highest risk of excess body weight occurs in children of mothers with pre-pregnancy obesity who were never breastfed, suggesting that breastfeeding may mitigate part of the intergenerational risk [226].

Although women with overweight or obesity are at greater risk of breastfeeding difficulties, they should be actively supported and encouraged to breastfeed. Targeted interventions—including counseling, structured follow-up, and, when needed, access to donor human milk—may help overcome barriers and reduce the risk of intergenerational obesity [227,228].

## 9. Conclusions

Maternal overweight and obesity are rising globally and are strongly linked to adverse outcomes for both mother and child. Alterations potentially caused by overweight and obesity in adipokines levels can contribute to inflammation, insulin resistance, and impaired maternal–fetal adaptations. Breast milk adipokines also represent an important pathway for early-life metabolic programming, potentially influencing long-term offspring health. The most relevant adipokines implicated in these alterations include leptin, adiponectin, ghrelin, resistin, and possibly obestatin.

Leptin regulates appetite, metabolism, reproduction, and pregnancy adaptation, with levels dependent on fat mass. During pregnancy, leptin—primarily of placental origin—increases and is higher in overweight/obese women, linking to GWG and preeclampsia. During lactation, it is present in breast milk, with the highest concentrations in colostrum, and reflects maternal adiposity; its levels are further modulated by maternal diet and postpartum weight changes, with potential effects on infant metabolic programming.

Adiponectin enhances insulin sensitivity and reproductive function but decreases in obesity and in late pregnancy. Low levels in overweight/obese women are associated with preeclampsia and fetal overgrowth. In breast milk, it declines as lactation progresses, with inconsistent associations with maternal BMI.

Ghrelin stimulates appetite and regulates energy balance. Its concentrations decrease in obesity and decline across pregnancy, peaking in mid-gestation. In breast milk, ghrelin levels are higher than in maternal serum and rise postpartum. Stress and GWG may influence pregnancy concentrations, but associations with maternal BMI remain inconsistent.

Resistin, a pro-inflammatory adipokine, promotes insulin resistance and lipid accumulation. It rises in late pregnancy, correlates with gestational age and cord blood levels, and may be partly regulated by the placenta. In breast milk, resistin peaks in the early postpartum period and subsequently declines, correlating with maternal serum levels, although its association with maternal BMI is inconsistent.

Obestatin, a peptide with opposite functions to ghrelin, shows altered levels in obesity; however, evidence linking maternal BMI with obestatin during pregnancy or lactation remains limited.

Future research should aim to clarify the causal pathways and evaluate targeted interventions to optimize maternal–infant metabolic health through modulation of adipokine profiles.

## Figures and Tables

**Table 1:** Reported alterations in adipokine profiles in relation to maternal overweight and obesity during pregnancy and lactation (OW = overweight, OB = obese, NW = normal weight).

Adipokine	Biological Fluid	Alterations	References	Findings
leptin	maternal serum	increased	Misra et al. [29]	1.8× higher in OW/OB vs. NW (per kg body mass, early pregnancy)
			Zielinska-Pukos et al. [30]	1.4× higher in OW and 4.5× higher in OB vs. NW (during breast feeding)
			Maple-Brown et al. [31]	Strong positive association with pre-pregnancy BMI (r = 0.54, *p* < 0.0001) in 2nd trimester
			Poniedziałek-Czajkowska et al. [32]	Higher in OW/OB vs. NW at 24–34 weeks (43.44 ± 31.41 vs. 21.29 ± 12.67 ng/mL, *p* = 0.0001)
			Ozias et al. [33]	OW/OB vs. NW: 66.3 ± 34.2 vs. 35.7 ± 19.3 ng/mL (*p* < 0.001); correlated with total fat mass (r = 0.782, *p* < 0.001)
	maternal plasma	increased	Karowicz-Bilińska et al. [34]	Positive correlation with BMI in OB vs. NW women at 20–24 weeks (*p* = 0.008)
			Malti et al. [35]	~40% higher in OB vs. NW across all trimesters
			Kugananthan et al. [36]	Positively correlated with maternal fat mass percentage in both whole and skim milk (*p* = 0.008; *p* = 0.007)
	breast milk	increased	Zielinska-Pukos et al. [30]	OB: up to 6.2× higher vs. NW
			Sadr Dadres et al. [37]	Positively associated with pre-pregnancy BMI (β = 0.525 crude; β = 0.494 adjusted; *p* < 0.001). Excessive gestational weight gain independently associated (adjusted β = 0.298; *p* = 0.009)
			Zamanillo et al. [38]	2.8-fold higher in OW/OB vs. NW at 1 month postpartum (*p* < 0.05); levels declined in NW but remained stable in OW/OB
			Fields et al. [39]	OW mothers: +96.5%; OB mothers: +315.1% vs. NW
adiponectin	maternal serum	decreased	Vernini et al. [40]	Negatively correlated with gestational BMI (r = −0.29, *p* = 0.013)
	maternal plasma	decreased	Nien et al. [41]	Median levels in OW vs. NW (7.40 [2.76–22.38] vs. 8.87 [2.77–25.03] mg/L; *p* < 0.05) (pregnant women)
			Suto et al. [42]	OW/OB vs. lean, 1st and 2nd trimester
		no correlation	Ozias et al. [33]	No difference between NW and OW/OB in 3rd trimester
	breast milk	decreased	Tekin Guler et al. [43]	Post-feed levels 12.84 ± 2.33 ng/mL (OB—pre-pregnancy BMI) vs. 13.95 ± 0.25 ng/mL (NW); *p* = 0.010
			Clark et al. [44]	NW: 12.35 ng/mL vs. OB: 8.70 ng/mL; *p* = 0.052
			Yu et al. [45]	β = 0.06; 95% CI: 0.02 to 0.10; *p* = 0.001; samples collected on days 3, 42, and 90 postpartum
		increased	Martin et al. [46]	β = 0.08 ± 0.02; *p* < 0.0001 (longitudinal data)
		no correlation	Christensen et al. [47]	Samples collected at three postpartum visits (1–8.49 months)
			Chan et al. [48]	Samples collected at 4 months postpartum.
			Sadr Dadres et al. [37]	Samples collected at 1 and 3 months postpartum
ghrelin	maternal plasma	no correlation	Tehranian et al. [49]	No significant difference between OW and NW from 1st to 2nd trimester (*p* > 0.05)
		increased	Aydin et al. [50]	Increase with postpartum weight loss, samples collected on days 1, 7, and 15 postpartum
	breast milk	increased	Tekin Guler et al. [43]	Higher in OB (pre-pregnancy BMI, *p* = 0.025); levels decreased over lactation but remained consistently elevated in OB
			Aydin et al. [50]	r = 0.42, *p* = 0.19; samples collected on days 1, 7, and 15 postpartum
		decreased	Yu et al. [45]	β = −0.08; 95% CI: −0.10 to −0.06; *p* < 0.001, samples collected on days 3, 42, and 90 postpartum
		no correlation	Andreas et al. [51]	At 1 week and 3 months postpartum, with BMI at sampling
obestatin	breast milk	decreased	Badillo-Suárez et al. [52]	Lower concentrations at 3–7, 14–15, and 30 days postpartum (adjusted *p* < 0.001) of mothers with higher body fat percentage
resistin	maternal serum	Increased	Anggraini et al. [53]	Median levels: 1.41 (1.02–1.95) ng/mL in OB vs. 1.31 (0.74–1.52) ng/mL in NW (*p*< 0.05)
		no correlation	Vernini et al. [40]	At 37–38 weeks of gestation, with gestational BMI assessed
	maternal plasma	increase	Ozias et al. [33]	No difference between NW (7.6 ± 2.9 ng/mL) and OW/OB (7.6 ± 4.3 ng/mL); *p* = 0.001. Positively correlated with visceral/total fat ratio (*p* = 0.045)
	breast milk	no correlation	Andreas et al. [51]	At 1 week and 3 months postpartum, with BMI at sampling
			Santosa et al. [54]	At 1 month postpartum, with maternal BMI assessed pre-pregnancy, at delivery, and at 1 month postpartum

## Data Availability

The data can be shared upon request.
